# Galactosyl BODIPY-based nanoparticles as a type-I photosensitizer for HepG2 cell targeted photodynamic therapy†

**DOI:** 10.1039/d4ra00041b

**Published:** 2024-03-14

**Authors:** Jin-Yu Liu, Ye Tian, Lei Dong

**Affiliations:** a School of Chemical Engineering, Lanzhou University of Arts and Science Lanzhou 730000 Gansu P. R. China 3153634066@qq.com; b School of Pharmacy, Shandong Second Medical University 7166 Baotong West St Weifang 261053 P. R. China leidong@wfmc.edu.cn; c Shandong Provincial No. 4 Institute of Geological and Mineral Survey 2375 Xiangyang Rd Weifang 261053 P. R. China tianye1469@163.com

## Abstract

We report a galactosyl diiodo-BODIPY-based nanoparticles as type-I photosensitizer (PS) with high water solubility for HepG2 cell targeted photodynamic therapy. Functionalized galactoside and glucoside were introduced into diiodo-BODIPY to obtain BP1 and BP2, respectively. The glycolyl PSs could self-assemble to form the nanoparticles BP1-NP and BP2-NP with red-shifted near-infrared (NIR) absorption and fluorescence at 682 nm and 780 nm, as well as excellent chemo- and photo-stability. In comparison to the monomer in DMSO, the aggregated photosensitizers in the nanoparticles enabled the sensitization of oxygen to superoxide (O_2_˙^−^) through a type-I process, while repressing the generation of singlet oxygen (^1^O_2_) through a type-II process. The galactosyl-modified BP1-NPs could target and concentrate on HepG2 cells, subsequently generating O_2_˙^−^ and ^1^O_2_ to trigger cell death under 660 nm light irradiation. This work provides an efficient strategy for the construction of glycoside-recognized type-I photosensitizers for tumor cell imaging and photodynamic therapy.

## Abstract 2

Herein, we report galactosyl diiodo-BODIPY-based nanoparticles as a type-I photosensitizer (PS) with high water solubility for HepG2 cell targeted photodynamic therapy.

## Graphical abstract

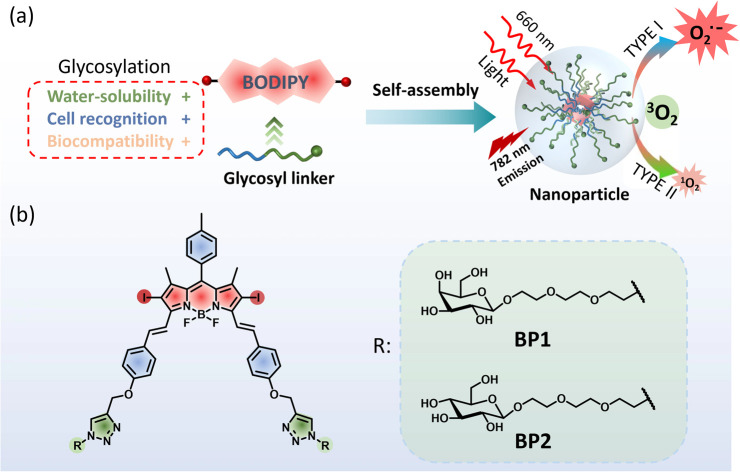

## Introduction

Photodynamic therapy (PDT), an innovative therapeutic modality, has attracted much attention for diverse treatments such as cancer, bacterial infections, and non-malignant diseases.^1–3^ Different from the common treatment methods (surgery, radiotherapy, and chemotherapy), the PDT strategy mainly depends on three crucial factors: photosensitizer (PS), oxygen, and light.^4^ The photosensitizer is noncytotoxic in a dark environment, but it can be activated by a particular light; this then sensitizes the surrounding oxygen to generate reactive oxygen species (ROS), which have cytotoxic effects, leading to cellular or bacterial apoptosis or necrosis. ROS are generated by excited PSs through two processes called type-I and type-II processes.^5^ First, PSs are irradiated under light to reach a specific excited singlet state (^1^PS*) and then undergo the intersystem crossing (ISC) process to an excited triplet state (^3^PS*). In the type-II process, a triplet–triplet energy transfer occurs between ^3^PS* and oxygen to generate the active singlet oxygen (^1^O_2_).^6–8^ In contrast, the type-I process involves a cascade of electron or proton transfer between the ^3^PS* and adjacent oxygen to afford superoxide (O_2_˙^−^), hydroxyl radicals (OH˙), or other ROS (Fig. S1†).^9,10^ However, the ^1^O_2_-generation efficiency of type-II PSs is dependent on the oxygen concentration, which is restricted for treatment in a hypoxic microenvironment. Type-I PSs are reported to reduce the oxygen requirement by avoiding O_2_ depletion in the PDT process, to overcome the hypoxic shortage, and thus they have become more welcomed in PS design and further PDT applications.^11^

In the past decades, there has been a rapid development of organic photosensitizers, including small organic PSs (porphyrins, phthalocyanines, indocyanine), metal complexes, and organic frameworks.^12^ Among the numerous small organic PSs, boron dipyrromethene (BODIPY) and its derivatives have emerged as a popular choice because of their outstanding photophysical and chemical properties, such as high molar extinction coefficient and fluorescence quantum yield, stable chemo- or photo-stability, and multiple modification capacity.^13^ Jiao's group enriched many BODIPY derivatives for biological imaging and PDT.^14–16^ Yang's group developed multiple BODIPY-based supramolecular systems for efficient type-I PDT.^17–19^ In addition, the light wavelength is equally important for the PDT process. Exciting light with a longer wavelength enables overcoming the self-absorption and scattering, which can increase the light-penetration depth. Therefore, it is of much significance to discover novel PSs with high absorption in therapeutic windows (650–900 nm).

For efficient PDT, it is essential that PSs are highly concentrated at the targeted position (tumor or bacteria). Co-assembling or introducing surfactants, targeting groups with other water-soluble moieties, is a general approach to enhance the hydrophilicity and biocompatibility, which even endow tumor recognition abilities to PSs. Some glycoside-modified compounds have been reported to selectively recognize receptors on the cytomembrane, thereby allowing them to highly concentrate on specific tumors.^20–22^ The innovations of glycosyl-modified PSs are meaningful for achieving highly efficient PDT but have rarely been reported to date (Table S1†).

**Fig. 1:**
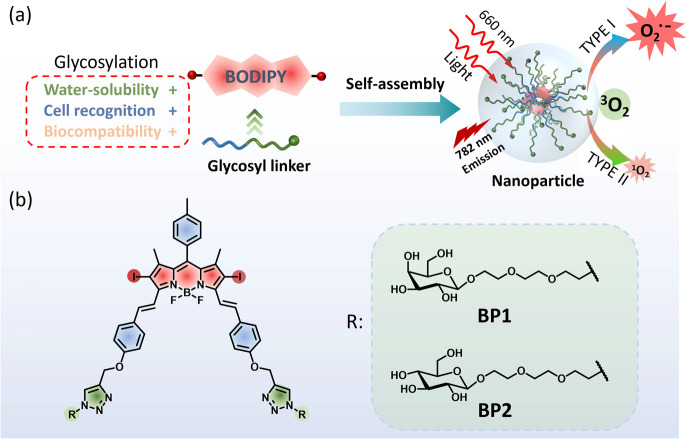
(a) Design of diglycosyl BODIPY PSs and the construction of nanoparticles for PDT. (b) Structures of galactosyl BODIPY (BP1) and glucosyl BODIPY (BP2).

Herein, we developed galactosyl diiodo-BODIPY-based nanoparticles as photosensitizers with a HepG2 cell targeting ability for NIR cell imaging and photodynamic therapy. Diiodo-BODIPY, as a classic PDT candidate, incorporated functionalized galactose (BP1) for the specific targeting of HepG2 cells and glucose (BP2) as a negative compound. Two glycosyl PSs were developed (BP1-NP and BP2-NP) that showed excellent water solubility and could self-assemble to form nanoparticles with about 100 nm hydrated size and homogenous morphologies. In comparison to the monomer (BP), the nanoparticles exhibit a red-shifted and broad absorption band and impaired fluorescence at 780 nm, which allowed them to be excited by near-infrared light. The nanoparticles could sensitize oxygen to mainly generate superoxide (O_2_˙^−^) in PBS buffer rather than singlet oxygen (^1^O_2_) in the monomer state in DMSO. BP1-NP could selectively recognize and concentrate in HepG2 cells, subsequently providing fluorescence signals, and exhibiting outstanding phototoxicity under 660 nm light irradiation but low cytotoxicity in dark environments (Fig. 1).

## Results and discussion

### Molecular design and synthesis

Coupling two or three BODIPY molecules, introducing a halogen atom (Br or I), and supramolecular self-assembly are popular strategies to construct BODIPY-based PSs.^23,24^ Diiodo-BODIPY is attracting much attention for photosensitizer design due to its high ROS generation efficiency, molar extinction efficiency, excellent chemo- and photo-stability, and multiple functionalized sites. The introduction of a phenyl moiety on the 3,5-site of BODIPY through a C=C linker can further red shift the absorption and fluorescence wavelengths, which is conducive to the photodynamic effect *in vivo*.^17,18^ It is well known that β-d-galactose has a high affinity to the ASGP receptor on the cytomembrane of hepatoma cells.^25^ Thus, triethylene glycol (TEG) functionalized galactose was introduced into the BODIPY core to enhance the cell targeting ability of BP1. Glucosyl BODIPY (BP2) was synthesized as a negative control in the meantime.

**Fig. 2:**
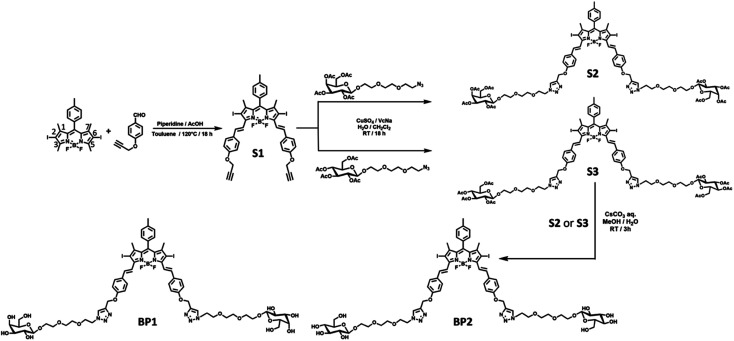
Synthesis scheme of galactosyl BODIPY (BP1), glucosyl BODIPY (BP2), and related derivatives.

Starting from the classic 2,6-diiodo-1,3,5,7-tetramethyl BODIPY,^26^ 4-propargyloxybenzadehyde^27^ was connected through a Knoevenagel condensation to obtain compound S1 with an extended π–π conjugation. Next, the two propargyl-modified S1 was decorated with two equivalent azido-TEG-galactose or glucose through a Cu(i)-catalysed cycloaddition reaction (“Click” reaction). The resulting acetyl-protected compounds S2 or S3 underwent solvolysis under a basic environment to afford the water-soluble photosensitizers BP1 or BP2, respectively (Fig. 2). The relevant characteristics obtained by ^1^H NMR, ^13^C NMR, high-resolution mass spectrometry (HRMS), and Fourier-transform infrared (FTIR) spectroscopy analyses are shown in the ESI.†

### Formation and properties of the nanoparticles

**Fig. 3:**
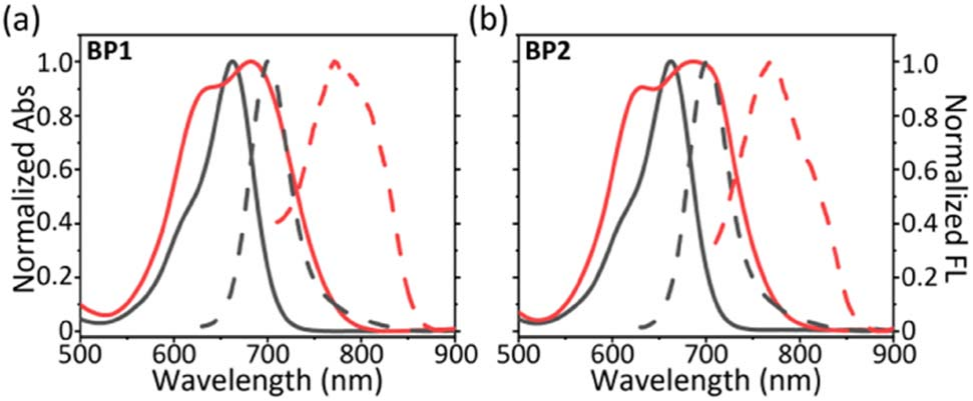
Normalized absorption (full line) and fluorescence (dash line) spectra of (a) BP1 and (b) BP2 in DMSO (monomer, grey) and in PBS buffer (aggregate, red).

With two glycosyl BODIPY PSs in hand, we evaluated their photophysical properties in organic solvents and aqueous solutions. With the assistance of two hydrophilic glycosides, BP1 and BP2 exhibited excellent water solubility, leading the two molecules to dissolve well in high-polar organic solvents, such as MeOH, DMF, and DMSO. Thus, we measured the photophysical properties of BP1 and BP2 in DMSO. BP1 and BP2 showed a single absorption peak at 662 nm with a molar extinction efficient (*ε*) as 8.7 × 10^4^ M^−1^ cm^−1^ (Fig. 3a and b, grey line). The fluorescence emission of BPs was at 700 nm with a sharp emissive band and low intensity (*Φ*_F_ = 3.1%). The similar photophysical properties of BP1 and BP2 were attributed to the flexible linker between the glycoside and BODIPY core, which might not influence the optical properties of the chromophores. However, the two glycosides could not provide sufficient water solubility to BP1 and BP2, resulting in the occurrence of self-assembly to obtain aggregated nanoparticles (BP1-NP and BP2-NP). In comparison to the monomer, the BP-NPs displayed dual-humped absorption at 632 nm and 682 nm (*ε* = 5.4 × 10^4^ M^−1^ cm^−1^), following impaired fluorescence at 780 nm (Fig. 3a and b, red line). The broad absorption band and quenched fluorescence were probably due to intermolecular disordered stacking. However, the absorption of BP1-NP and BP2-NP showed only a slight variation over 14 days at room temperature (RT), indicating that the nanoparticles have sufficient structural stability to disperse in PBS buffer (Fig. S2†).

**Fig. 4:**
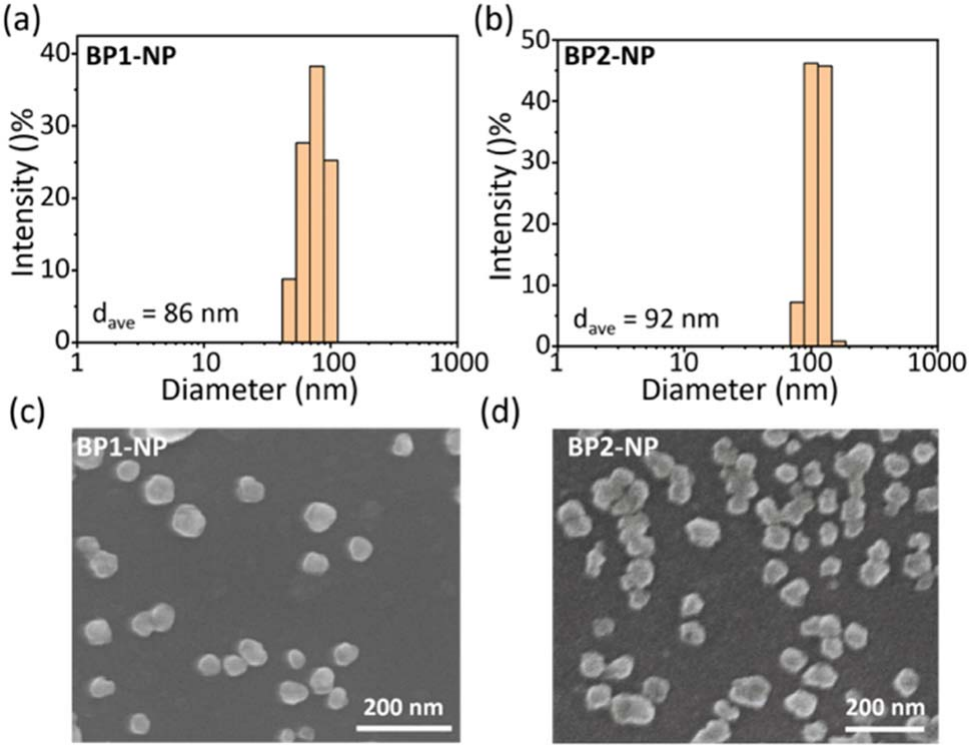
Dynamic laser scattering measurements of the average hydrated sizes of the nanoparticles (a) BP1-NP and (b) BP2-NP in PBS buffer (*c* = 50 μM, pH 7.4). Scanning electron microscopy images for the morphology imaging of (c) BP1-NP and (d) BP2-NP.

Next, we explored the hydrated size and morphology of the two nanoparticles. Concentrated DMSO solutions of BP1 or BP2 were added to PBS buffer, followed by vigorous stirring for 1 h to obtain the corresponding nanoparticles. Dynamic laser scattering (DLS) tests evaluated the average hydrated size of BP1-NP as *ca.* 86 nm (Fig. 4a) and BP2-NP as *ca.* 92 nm (Fig. 4b), respectively. The results indicated that the hydrophilicity provided by TEG-glycosides could sufficiently keep the nanoparticles well dispersed in PBS buffer without any surfactant intervention needed. In addition, scanning electron microscopy (SEM) was used to investigate the morphologies of BP1 and BP2 (Fig. 4c and d), indicating that both nanoparticles have irregular nubbly morphology. The homogenous sizes of BP1 and BP2 from SEM were in agreement with the narrow distribution from the DLS data. The subtle difference between BP1 and BP2 might be ascribed to the conformation of the glycosides (Gal or Glc), which slightly influence the water solubility and molecule aggregate.

### ROS generation and stability

**Fig. 5:**
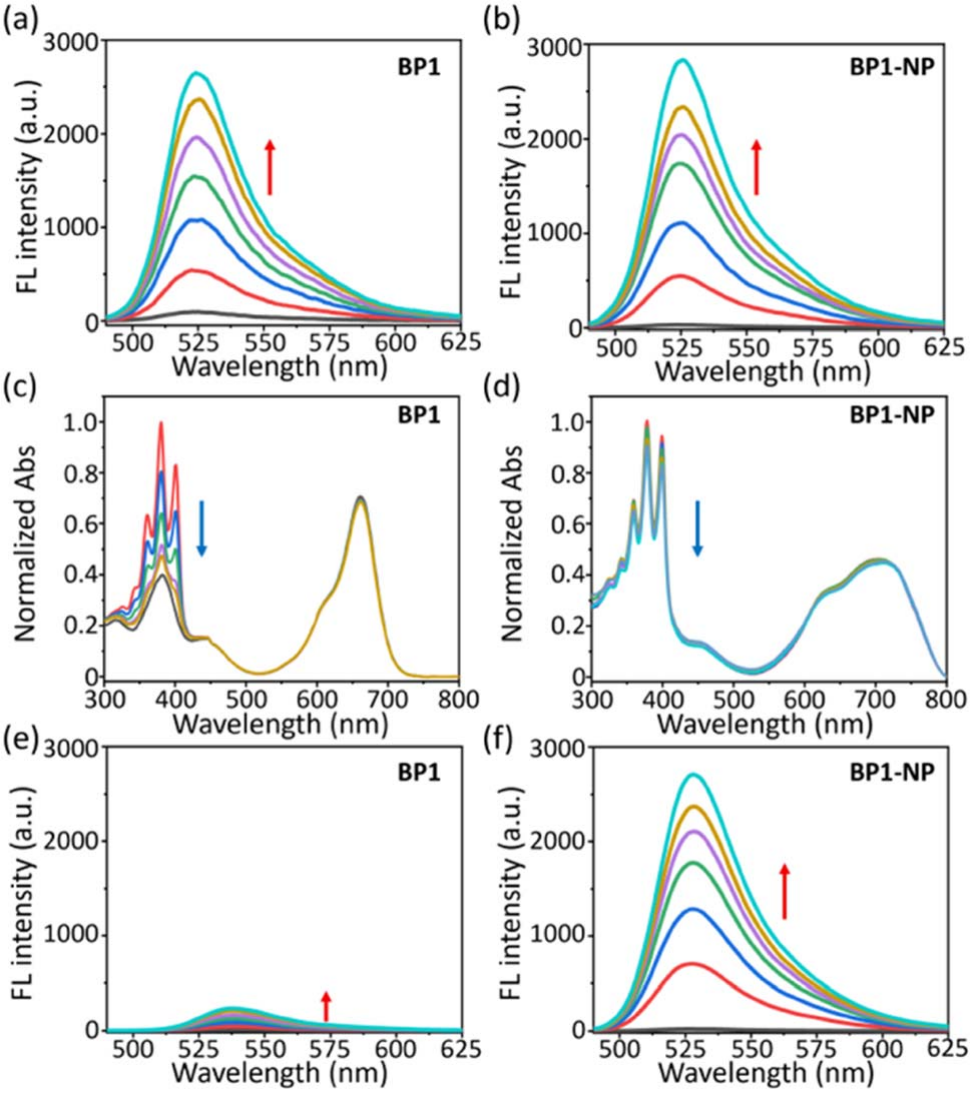
Fluorescence spectra of DCHF in the (a) DMF solution of BP1 or (b) PBS dispersion of BP1-NP under 660 nm light irradiation for 3 min (interval 30 s, *P* = 50 mW cm^−2^, *λ*_ex_ (DCFH) = 470 nm). (c) Absorption spectra of ABDA in the (c) DMF solution of BP1 or (d) PBS dispersion of BP1-NP under 660 nm light irradiation for 3 min (interval 30 s, *P* = 50 mW cm^−2^). Fluorescence spectra of DHR 123 in the (e) DMF solution of BP1 or (f) PBS dispersion of BP1-NP under 660 nm light irradiation for 3 min (interval 30 s, *P* = 50 mW cm^−2^, *λ*_ex_ (DHR 123) = 470 nm).

We evaluated the ROS generation ability of the glycosyl BODIPY PSs in the monomer and nanoparticle state. Due to the same BODIPY core of BP1 and BP2, we only measured the ROS generation of BP1. First, the ROS generation ability of BP1 in monomer (DMF) and aggregated (BP1-NP in PBS) states were measured using 2′,7′-dichlorofluorescein (DCFH, a commercial ROS probe). Under 660 nm light irradiation (*P* = 50 mW cm^−2^), an enhanced fluorescence of DCFH at 525 nm was observed upon increasing the irradiation time, indicating that BP1 could generate ROS in both the monomer and aggregated states (Fig. 5a and b). To further investigate the types of ROS generated, we evaluated the singlet oxygen (^1^O_2_) generation ability of BP1 in DMF or PBS buffer under light irradiation (*P* = 50 mW cm^−2^). Herein, 9,10-anthracenediyl-bis(methylene)dimalonic acid (ABDA) was used as a typical ^1^O_2_ capture agent whose symbolic triplet absorption peaks at 380 nm was significantly quenched in the presence of ^1^O_2_. When turning on the red-light source (660 nm), the ABDA absorption in the DMF solution of BP1 rapidly decreased over 2 min. The results indicated that BP1 could efficiently generate ^1^O_2_ in the monomer state, which is in accordance with the reference (Fig. 5a and S3a†).^28^ The nanoparticle BP1-NP in PBS was similarly exposed to light irradiation, and the nanoparticles were observed to generate ^1^O_2_ in PBS buffer; however, the absorption quenching of ABDA became distinctly slower than in DMF, revealing that the sensitization ability of ^1^O_2_ was disturbed in the aggregate state (Fig. 5b and S3a†). In consideration of the excellent ROS generation results of BP1-NP (Fig. 5b), we speculated that the nanoparticles may generate other ROS instead of ^1^O_2_ under light irradiation. Hence, we monitored the superoxide (O_2_˙^−^) generation of BP1-NP using dihydrorhodamine 123 (DHR123), which can “turn-on” the fluorescence at 530 nm after responding to reactive oxide radical species. Different from the feeble emission in DMF of BP1, a fast fluorescence enhancement of DHR 123 in the PBS dispersion of BP1-NP was monitored (Fig. 5e and f). To further confirm the results, we used dihydroethidium (DHE) to detect the generated O_2_˙^−^ in a PBS dispersion of BP1-NP, in which fluorescence at 580 nm can be turned on after capturing O_2_˙^−^. In the dark environment, the DHE maintained a quenched fluorescence in BP1-NP solution, but when exposed to 660 nm light irradiation, the fluorescence enhancement of DHE could be clearly observed, indicating the generation of O_2_˙^−^ by the nanoparticles (Fig. S3b†). Thus, the nanoparticles self-assembled from BP1 or BP2 might play the role of type-I and type-II photosensitizers in PBS buffer. Moreover, BP1-NP and BP2-NP exhibited outstanding photo- and chemo-stability (Fig. S4 and S5†), whose absorption showed negligible variation whether exposed to light irradiation (50 mW cm^−2^) or the presence of H_2_O_2_ (100 μM). The satisfactory ROS generation of BP-NP suggests its potential for PDT application in cells.

### Photodynamic therapy in cells

**Fig. 6:**
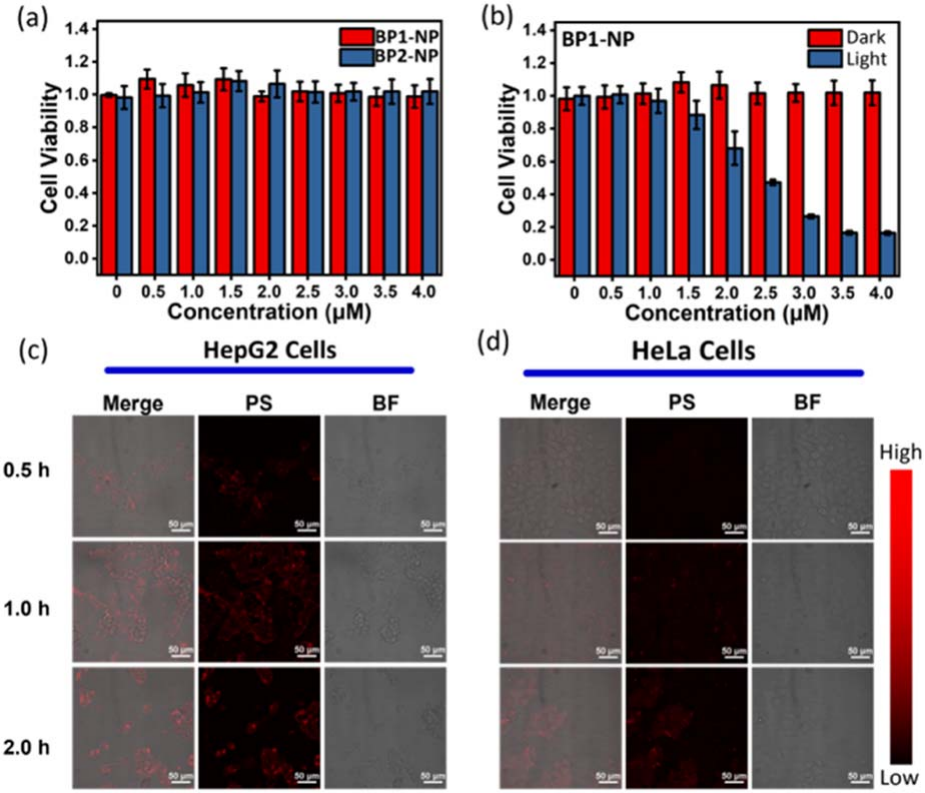
(a) Cell viability of HepG2 cells after incubating with BP1-NP or BP2-NP with different concentrations (0–4 μM) for 24 h. (b) Cell viability of HepG2 cells incubated with different concentrations (0–4 μM) of BP1-NP in a dark environment or exposed to 660 nm light irradiation (*P* = 50 mW cm^−2^). Laser confocal microscope imaging of (c) HepG2 cells and (d) HeLa cells after incubation with BP1-NP for different times (*c* = 4 μM).

The nanoparticles BP1-NP and BP2-NP were used to evaluate their potential for photodynamic therapy in cells. Cell viability in a dark environment was first evaluated at different concentrations (0–4.0 μM) over 24 h, and the results indicated that the glycosyl-modified nanoparticles BP1-NP and BP2-NP have low cytotoxicity for cell proliferation (Fig. 6a). Next, the cell uptake of the two nanoparticles were investigated. After incubating the dispersion of BP1-NP or BP2-NP, a gradually enhanced fluorescence imaging of HepG2 cells was captured during 0–3 h, indicating that the glycosyl aza-BODIPY-based nanoparticles could provide a sufficient fluorescence signal for bioimaging *in vitro* (Fig. 6c and S6a†). While incubating with HeLa cells, BP1-NP provided a negligible fluorescence signal while BP2-NP could still distinctly mark the cells (Fig. 6d and S6b†). The difference in the imaging ability of BP1-NP towards HepG2 or HeLa cells suggested that the recognition between galactoside and HepG2 cells influenced the cellular endocytosis of BP1-NP.^29^ These results sufficiently demonstrated that galactosyl-modified BP1-NP could selectively mark and image HepG2 cells. Finally, HepG2 cells were incubated with different concentrations (0–4.0 μM) of BP1-NP for 2 h and then placed in a dark environment or exposed to 660 nm laser irradiation for 15 min (*P* = 50 mW cm^−2^). The cell viability showed no change in the dark environment but exhibited a gradual decrease under light irradiation following the increase in concentration of nanoparticles. BP1-NP showed outstanding phototoxicity and could decrease the cell viability to less 20% under a low concentration level (4 μM). After incubating with BP1-NP (4 μM), a persistent decrease in cell viability was observed with increasing the time of light irradiation (Fig. S7†). Thus, the galactosyl-modified photosensitizer BP1-NP could provide dual functions of fluorescence imaging and photodynamic therapy towards HepG2 cells.

## Conclusion

In summary, we developed two glycosyl diiodo-BODIPY-based photosensitizers with high water solubility, biocompatibility, and cell targeting ability for photodynamic therapy. Based on 2,6-diiodo-BODIPY, TEG-linked galactose was introduced on the chromophore to obtain BP1 with a HepG2 cell targeting ability. Glucosyl-modified BP2 was used as the negative control. BP1 and BP2 self-assembled to form nanoparticles with a red-shifted absorption band at 682 nm (*ε* = 5.4 × 10^4^ M^−1^ cm^−1^) and quenched fluorescence emission. The nanoparticles BP1-NP and BP2-NP possessed homogenous irregular morphology with hydrated sizes of 86 and 95 nm, respectively. In comparison to the monomer of BP1, the aggregated BP1-NP has O_2_˙^−^ generation ability but repressive ^1^O_2_ generation ability in PBS buffer. The two nanoparticles exhibited outstanding stability in room temperature, light irradiation, and oxidation environments. Due to the galactosides, BP1-NP could specifically recognize and concentrate on HepG2 cells, providing a fluorescence signal and inducing cell death under 660 nm light irradiation. We not only developed a series of glycosyl BODIPY photosensitizers for photodynamic therapy but also provide another design strategy for the improvement of the cytotoxicity and cell targeting ability of photosensitizers.

## Conflicts of interest

There are no conflicts to declare.

## Supplementary Material

RA-014-D4RA00041B-s001
