# Supplementary material for: Galactosyl BODIPY-based nanoparticles as a type-I photosensitizer for HepG2 cell targeted photodynamic therapy
Source: RSC Adv. 2024 Mar 14;14(13):8735–9. doi: 10.1039/d4ra00041b (PMC10938552; doi:10.1039/d4ra00041b)
Supplement: RA-014-D4RA00041B-s001 [file RA-014-D4RA00041B-s001.pdf]

Electronic Supplementary Material (ESI) for RSC Advances.
This journal is © The Royal Society of Chemistry 2024

Supporting Information

Galactosyl BODIPY-based Nanoparticles as Type-I Photosensitizer for HepG2 Cell Targeted Photodynamic Therapy

Jin-Yu Liu,[a] Ye Tian[c] and Lei Dong*[b]

[a] School of Chemical Engineering, Lanzhou University of Arts and Science, Lanzhou, 730000, Gansu, P.R. China.

[b] School of Pharmacy, Shandong Second Medical University, 7166 Baotong West St., Weifang 261053, P.R. China.

[c] Shandong Provincial NO. 4 Institute of Geological and Mineral Survey, 2375 Xiangyang Rd., Weifang 261053, P.R. China.

* Corresponding author: leidong@wfmc.edu.cn

Contents List

Synthesis of photosensitizers **BP1** and **BP2** S1-S4

Experimental section S5-S6

Additional figures and schemes S7-S15

References S15

Synthesis of photosensitizers BP1 and BP2

General methods

All reagents for synthesis commercially available (highest purity available for reagent grade compounds) were used without further purification. Reactions under microwave activation were performed on a Biotage Initiator system. Thin-layer chromatography (TLC) was carried out on aluminum sheets coated with silica gel 60 F_{254} (Merck). TLC plates were inspected by UV light (λ = 254 nm, 365 nm) and developed by treatment with a mixture of 10% H_2SO_4 in EtOH/H_2O (95:5 v/v) followed by heating. Silica gel column chromatography was performed with silica gel Si 60 (40–63 µm). NMR spectra were recorded at 293 K, unless stated otherwise. Chemical shifts are referenced relative to deuterated solvent residual peaks. The following abbreviations are used to explain the observed multiplicities: s, singlet; d, doublet; t, triplet; q, quadruplet; m, multiplet; p, pseudo and b, broad. High resolution (HR-ESI-QToF) mass spectra were recorded using a Bruker MicroToF-Q II XL spectrometer.

Synthesis procedures

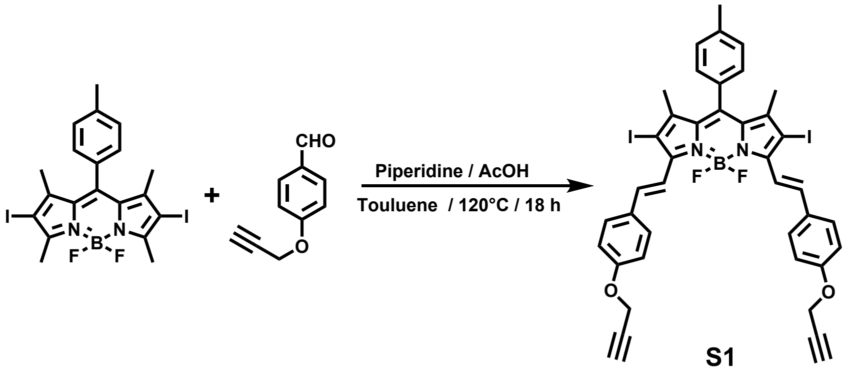

*Synthesis procedure of compound **S1***:

According to the reported method[1], to a solution of 2,6-diiodo-1,3,5,7-tetramethyl BODIPY[2] (1 g, 1.69 mmol, 1 eq.) and 4-propargyloxybenzaldehyde[3] (814 mg, 5.08 mmol, 3 eq.) in dry toluene was added piperidine (1 mL) and acetic acid (AcOH, 0.5 mL). The resulting mixture was bubbled under N_2 during 10 min, and then heated at 120°C during 18 h. After cooling to room temperature (RT), the reaction was diluted with CH_2Cl_2 (100 mL), washed with HCl aqueous solution (30 mL ◊2) and brine (50 mL). The combined organic layer was dried (Na_2SO_4), concentrated and purified with silica gel column chromatography (Cyclohexane:CH_2Cl_2 = 1:3) to afford compound **S1** (945 mg, 64%) as a dark purple powder.

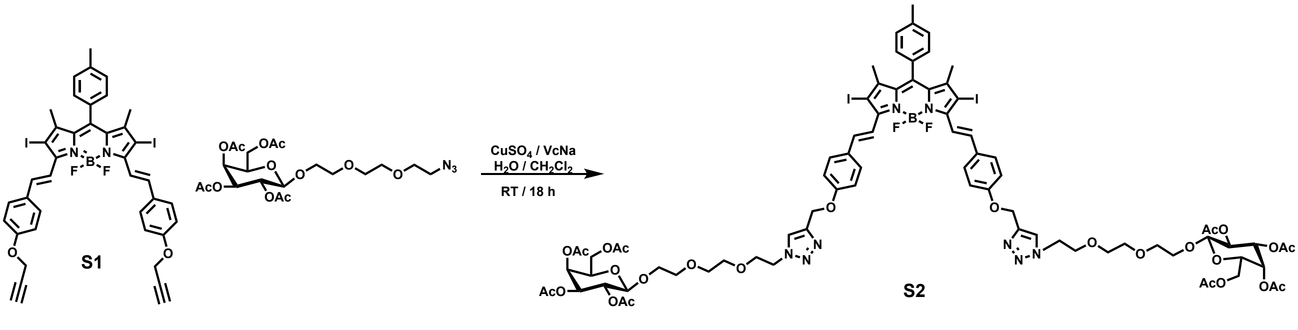

*Synthesis procedure of compound **S2***:

To a solution of **S1** (400 mg, 0.46 mmol, 1 eq.) and prefabricated azido-TEG-galactose[4] (694 mg, 1.37 mmol, 3

eq.) in CH_2Cl_2 (20 mL) was added copper sulfate ($CuSO_4$, 37 mg, 0.23 mmol, 0.5 eq.), sodium ascorbate (VcNa, 272 mg, 1.37 mmol, 3 eq.) and diluted water (10 mL). The resulting mixture was vigorously stirred at RT for 24 h under TLC monitoring the disappearance of **S1**. The reaction was diluted with (50 mL), washed with saturated edetate disodium (EDTA-Na_2, 50 mL◊2), and brine (50 mL). The combined organic layer was dried (Na_2SO_4), concentrated and purified with silica gel column chromatography (CH_2Cl_2:MeOH =50:1) to afford compound **S2** (692 mg, 80%) as an dark purple amorphous solid .

^{1}H NMR (600 MHz, $CDCl_3$): δ (ppm) 8.11 (d, 2H, J = 16.7 Hz), 7.84 (s, 2H), 7.61 (d, 4H, J = 7.9 Hz), 7.58 (d, 2H, J = 16.7 Hz), 7.33 (d, 2H, J = 7.9 Hz), 7.16 (d, 2H, J = 8.0 Hz), 7.06 (d, 4H, J = 8.8 Hz), 5.39 (dd, 2H, J = 1.0, 3.4 Hz), 5.29 (s, 4H), 5.19 (dd, 2H, J = 8.0, 10.5 Hz), 5.01 (dd, 2H, J = 3.5, 10.5 Hz), 4.57 (t, 4H, J = 5.1 Hz), 4.52 (d, 2H, J = 8.0 Hz), 4.10-4.17 (m, 8H), 3.93-3.97 (m, 2H), 3.87-3.90 (m, 6H), 3.69-3.71 (m, 2H), 3.58-3.61 (m, 12H), 2.47 (s, 3H), 2.13 (s, 6H), 2.029 (s, 6H), 2.025 (s, 6H), 1.97 (s, 6H), 1.47 (s, 6H).

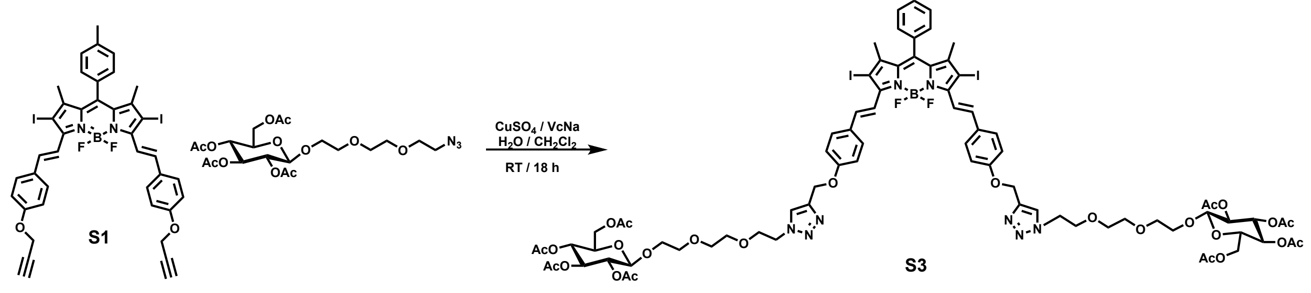

*Synthesis procedure of compound **S3***:

To a solution of **S1** (410 mg, 0.47 mmol, 1 eq.) and prefabricated azido-TEG-glucose[4] (711 mg, 1.41 mmol, 3 eq.) in CH_2Cl_2 (20 mL) was added copper sulfate ($CuSO_4$, 38 mg, 0.23 mmol, 0.5 eq.), sodium ascorbate (VcNa, 279 mg, 1.41 mmol, 3 eq.) and diluted water (10 mL). The resulting mixture was vigorously stirred at RT for 24 h under TLC monitoring the disappearance of **S1**. The reaction was diluted with (50 mL), washed with saturated edetate disodium (EDTA-Na_2, 50 mL◊2), and brine (50 mL). The combined organic layer was dried (Na_2SO_4), concentrated and purified with silica gel column chromatography (CH_2Cl_2:MeOH =50:1) to afford compound **S3** (647 mg, 72%) as an dark purple amorphous solid .

^{1}H NMR (600 MHz, $CDCl_3$): δ (ppm) 8.11 (d, 2H, J = 16.6 Hz), 7.84 (s, 2H), 7.60 (d, 4H, J = 8.8 Hz), 7.57 (d, 2H, J = 16.7 Hz), 7.32 (d, 2H, J = 7.7 Hz), 7.15 (d, 2H, J = 8.0 Hz), 7.05 (d, 4H, J = 8.8 Hz), 5.34 (dd, 2H, J = 3.5, 10.1 Hz), 5.30 (s, 2H), 5.28 (s, 4H), 5.26 (dd, 2H, J = 1.7, 3.4 Hz), 4.86 (d, 2H, J = 1.8 Hz), 4.58 (t, 4H, J = 5.2 Hz), 4.52 (d, 2H, J = 8.0 Hz), 4.27 (dd, 2H, J = 5.0, 12.3 Hz), 4.08-4.13 (m, 4H), 4.03-4.05 (m, 2H), 3.9 (t, 4H, J = 5.4 Hz), 3.78-3.81 (m, 2H), 3.58-3.63 (m, 16H), 2.47 (s, 3H), 2.14 (s, 6H), 2.09 (s, 6H), 2.03 (s, 6H), 1.98 (s, 6H), 1.46 (s, 6H).

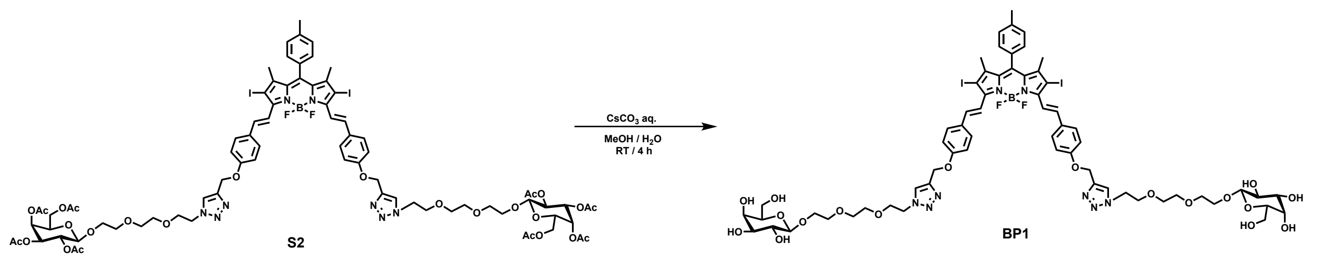

*Synthesis procedure of compound **BP1***:

To the solution of compound **S2** (150 mg, 0.079 mmol, 1 eq.) in MeOH (30 mL) with traced THF was added the solution of Cs_2CO_3 (311 mg, 0.95 mmol, 12 eq.) in H_2O (5 mL). The resulting solution was stirred at RT during 4 h until the deprotection was finished. The mixture was filtered under vacuum and the residues was sufficiently washed with acidic solution (20 mL◊2), diluted H_2O (20 mL◊2), MeOH (10 mL◊2) and THF (10 mL◊2), dried under vacuum to afford the targeting photosensitizer **BP1** (101 mg, 82%)as the amorphous dark purple solid.

^{1}H NMR (600 MHz, DMSO-d_6): δ (ppm) 8.25 (s, 2H), 8.07 (d, 2H, J = 16.5 Hz), 7.60 (d, 4H, J = 8.8 Hz), 7.42-7.45 (m, 4H), 7.33 (d, 2H, J = 8.0 Hz), 7.17 (d, 4H, J = 8.8 Hz), 5.23 (s, 4H), 4.84 (d, 2H, J = 4.6 Hz), 4.70 (d, 2H, J = 5.3 Hz), 4.54-4.58 (m, 6H), 4.37 (d, 2H, J = 4.6 Hz), 4.08 (d, 2H, J = 7.3 Hz), 3.81-3.84 (m, 6H), 3.61-3.63 (m, 2H), 3.47-3.52 (m, 20H), 3.23-3.27 (m, 4H), 2.44 (s, 3H), 1.43 (s, 3H).

^{13}C NMR (150 MHz, DMSO-d_6): δ (ppm) 159.5, 149.9, 145.6, 142.2, 139.5, 139.3, 138.7, 132.7, 131.4, 130.1, 129.0, 128.9, 128.1, 125.2, 116.2, 115.6, 103.6, 84.3, 75.2, 73.5, 70.5, 69.7, 69.6, 69.5, 68.7, 68.2, 67.7, 61.3, 60.5, 49.5, 21.1, 17.2.

HR-ESI-MS m/z: calcd. for $C_{64}H_{77}BF_2I_2N_8O_{18}$ $[M+H]^+$ 1548.3506, found 1549.3580.

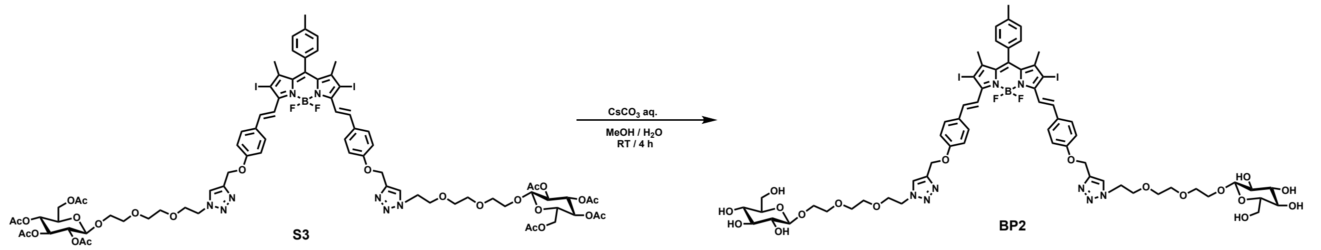

*Synthesis procedure of compound **BP2***:

To the solution of compound **S3** (100 mg, 0.053mmol, 1 eq.) in MeOH (30 mL) with traced THF was added the solution of Cs_2CO_3 (208 mg, 0.64 mmol, 12 eq.) in H_2O (5 mL). The resulting solution was stirred at RT during 4 h until the deprotection was finished. The mixture was filtered under vacuum and the residues was sufficiently washed with acidic solution (20 mL◊2), diluted H_2O (20 mL◊2), MeOH (10 mL◊2) and THF (10 mL◊2), dried under vacuum to afford the targeting photosensitizer **BP2** (61 mg, 74%)as the amorphous dark purple solid.

^{1}H NMR (600 MHz, DMSO-d_6): δ (ppm) 8.16 (s, 2H), 7.99 (d, 2H, J = 16.5 Hz), 7.51 (d, 4H, J = 7.3 Hz), 7.34-7.37 (m, 4H), 7.24 (d, 2H, J = 6.1 Hz), 7.09 (d, 4H, J = 7.3 Hz), 5.15 (s, 4H), 4.93 (s, 2H), 4.83-4.87 (m, 4H), 4.44-4.47 (m, 6H), 4.05 (d, 2H, J = 6.0 Hz), 3.75 (s, 6H), 3.56-3.59 (m, 2H), 3.41-3.44 (m, 16H), 2.87-3.05 (m, 8H), 2.36 (s, 3H), 1.35 (s, 6H).

^{13}C NMR (150 MHz, DMSO-d_6): δ (ppm) 159.7, 150.1, 145.9, 142.5, 139.8, 139.6, 139.0, 132.9, 131.6, 130.4, 129.2, 129.1, 128.4, 125.4, 116.4, 115.9, 103.2, 84.6, 77.1, 77.0, 73.6, 70.3, 69.9, 69.9, 69.8, 68.9, 68.1, 61.5, 61.3, 49.7, 21.3, 17.4.

HR-ESI-MS *m/z*: calcd. for $C_{64}H_{77}BF_2I_2N_8O_{18}$ $[M+H]^+$ 1548.3506, found 1549.3586.

Experimental section

Material and instruments. Materials were obtained from commercial suppliers and were used without further purification. All reactions were performed in oven-dried glassware (Hinwil) unless otherwise stated. Column chromatography was performed over silica gel (200-300 mesh). NMR spectra were recorded with JEOL-400 or JEOL-600 spectrometers. High-resolution mass spectrometry experiments were recorded by Bruker Solarix XR Fourier Transform Ion Cyclotron Resonance Mass Spectrometer. All optical spectra were recorded at room temperature. Absorption spectra of liquid samples were determined on Hitachi UV-3900 spectrophotometer. Fluorescence spectra of liquid samples were determined on determined on Hitachi F-4600 spectrophotometer. The absolute quantum yield was determined on Hamamatsu Quantaurus-QY. Dynamic light scattering (DLS) investigations were carried out with a DynaPro NanoStar dynamic light scattering detector. Scanning electron microscope (SEM) images were obtained using a Hitachi SU-8010 instrument. The photostability was conducted under irradiation with a high-power LED light and monitored by using an UV-3900 spectrophotometer. Confocal fluorescence imaging was performed with Nikon A1R MP multiphoton microscopy. Cell viability test was obtained on a Thermo Scientific Multiskan. Irradiation was performed by using a LED light (660 nm, PLS-LED 100, Perfect Light, Beijing, China).

Nanoparticle formation. Compound **BP1** or **BP2** (1 mmol) was well-dissolved in DMSO (20 μL). The resulting solution was added 9980 μL deionized (DI) water or PBS buffer (pH 7.4, 1 mM) and then vigorously stirred for 1 h at room temperature to form the nanoparticle **BP1-NP** or **BP2-NP** (100 μM). The nanoparticle dispersion in water or PBS buffer was diluted to low concentration for spectral measurements.

UV-Vis-NIR absorption. The UV-Vis-NIR absorption spectra were measured at room temperature using a Hitachi UV-3900 spectrophotometer. All spectra were corrected for background intensities by subtracting the spectra of pure solvent measured under identical conditions.

Fluorescence spectroscopy. The fluorescence measurements were carried out at room temperature using Hitachi F-4600 spectrophotometer. The fluorescence emission of **BP1** and **BP2** in monomer state was measured in DMSO (λ_{ex} = 610 nm, slit width 5-5 nm, 700 V). The fluorescence emission of **BP1-NP** and **BP2-NP** in aggregate state was measured in PBS buffer (λ_{ex} = 660 nm, slit width 10-20 nm, 700 V).

Dynamic light scattering. The dispersion of **BP1-NP** or **BP2-NP** in H_2O was diluted to 50 μM, and then measured the hydrated size by DynaPro NanoStar dynamic light scattering detector.

Detection of ROS production by DCFH in solution. Commercial probe 2',7'-dichlorofluorescein (DCFH) was used as ROS detector. 10 μM of **BP1** were dissolved in 2 mL DMF containing 20 μM of DCFH. 10 μM of **BP1-NP** were dissolved in 2 mL PBS buffer (7.4, 1 mM) containing 20 μM of DCFH. The mixture was then placed in a cuvette and irradiated with a 660 nm LED light at 50 mW cm^{-2}. The fluorescence change of sample at 525 nm was recorded under excitation at 470 nm.

Detection of $^1O_2$ production in solution. Compound 9,10-anthracenediyl-bis(methylene)-dimalonic acid (ABDA) was used as indicator for detection of $^1O_2$ in solution. When $^1O_2$ is generated in the system, the ABDA will be oxidized and the absorption at 380 nm decrease. 10 μM of **BP1** were dissolved in 2 mL DMF containing 50 μM of ABDA. 10 μM of **BP1-NP** were dissolved in 2 mL PBS buffer (7.4, 1 mM) containing 50 μM of ABDA. The mixture

was then placed in a cuvette and irradiated with a 660 nm LED light at 50 mW cm^{-2}. The absorption change of sample at 380 nm was recorded by the UV-Vis absorption spectrophotometer.

Detection of $O_2^{-•}$ production with DHR123 in solution. Commercial probe dihydrorhodamine 123 (DHR123) was used as $O_2^{-•}$ detector. 10 μM of **BP1** were dissolved in 2 mL DMF containing 20 μM of DHR123. 10 μM of **BP1-NP** were dissolved in 2 mL PBS buffer (7.4, 1 mM) containing 20 μM of DHR123. The mixture was then placed in a cuvette and irradiated with a 660 nm LED light at 50 mW cm^{-2}. The fluorescence change of sample at 530 nm was recorded under excitation at 470 nm.

Detection of $O_2^{-•}$ production with DHE in solution. Dihydroethidium (DHE) was used as indicator for detection of $O_2^{-•}$ in solution. When $O_2^{-•}$ is generated in the system, DHE can be oxidized to form ethidium which intercalates into DNA and emits bright fluorescence at ~580 nm. 10 μM of **BP1-NP** dispersion were dissolved in 2 mL solvent containing 30 μM of DHE and 200 μg/mL ctDNA. The mixture was then placed in a cuvette and irradiated with a 660 nm LED light at 50 mW cm^{-2}. The fluorescence change of sample was recorded by the fluorescence spectrometer.

Nanoparticle stability at room temperature. The prefabricated nanoparticle of **BP1-NP** (200 μL, 100 μM) was diluted in PBS buffer (pH 7.4, 1800 μL) to afford the diluted concentration (10 uM), and placed at room temperature. Absorption spectra were determined within 14 days (interval 2 d).

Nanoparticle photostability. The prefabricated nanoparticle of **BP1-NP** (200 μL, 100 μM) was diluted in PBS buffer (pH 7.4, 1800 μL) to afford the diluted concentration (10 uM). The resulting diluted nanoparticle dispersion in PBS buffer was exposed under 660 nm LED light irradiation (P = 50 mW cm^{-2}) during 2 h. Absorption spectra were determined within 2 h (interval 30 min).

Nanoparticle chem-stability in H_2O_2 solution. The prefabricated nanoparticle of **BP1-NP** (200 μL, 100 μM) was diluted in PBS solution (pH 7.4, 1960 μL) of H_2O_2 (1 mM) to afford the diluted concentration (10 uM). The resulting diluted nanoparticle dispersion in PBS buffer was determined absorption spectra variations within 2 h (interval 20 min).

***In vitro* cytotoxicity.** HepG2 or HeLa cells were seeded in 96-well plates (5×10^3 cells $well^{-1}$) and incubated for 24 h under normoxia. Then, the medium was replaced with 100 μL of DMEM (contain 5‰ DMSO) containing different concentrations (0-4 μM) of **BP1-NP** or **BP2-NP**. After incubation for another 120 min, the cells were washed three times with PBS, infused with fresh medium, and illuminated by a LED light (660 nm, 50 mW cm^{-2}) for 10 min. After further incubation for 24 h, the cell viability was examined by cell counting kit-8 (CCK-8) assays. Moreover, the dark toxicity of **BP1-NP** or **BP2-NP** were also analysed by the above procedure except the illumination was eliminated.

Additional figures and schemes

Table S1. A summary of reported photosensitizer for Type-I / II photodynamic therapy

No.	Structure	λ_{ex} / λ_{em} (nm)	ROS	Targeting	Reference
1	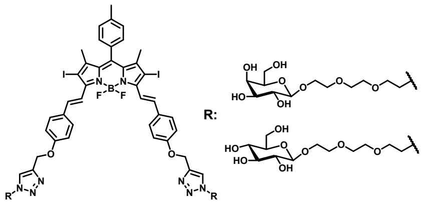	**682 / 780**	**$O_2^{\cdot-}$**	**Galactoside for HepG2 cell**	This work
2	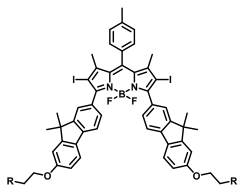	600 / 677	$O_2^{\cdot-}$ / OH·	--	*J. Am. Chem. Soc.* **2023**, *145*, 4081–4087
3	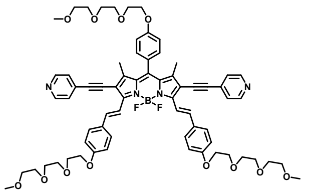	675 / 710	$^1O_2$	--	*ACS Materials Lett.* **2023**, *5*, 180–188
4	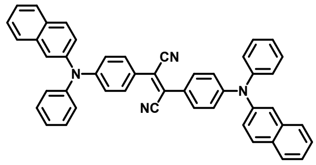	509 / 690	$^1O_2$	--	*Chem. Sci.*, **2023**,*14*, 684-690
5	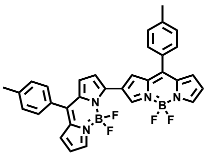	625/ 705	$O_2^{\cdot-}$	--	*Angew. Chem. Int. Ed.* **2021**, *60*, 19912-19920
6	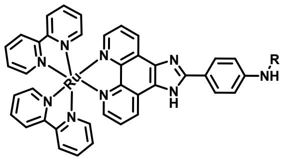	460 / 617	$^1O_2$	Mitochondria Targeting	*J. Am. Chem. Soc.* **2017**, *139*, 2512–2519
7	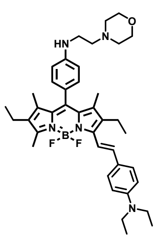	632 / 770	$^1O_2$	Lysosome Targeting	*Org. Biomol. Chem.* **2023**, *21*, 4672-4682
8	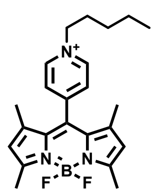	510 / 580	$O_2^{\cdot-}$	--	*Adv. Healthcare Mater.* **2023**, *12*, 23010

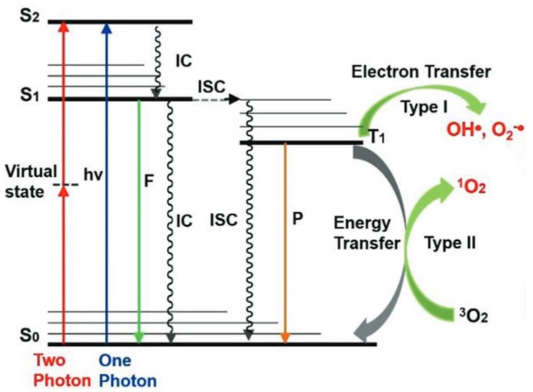

Figure S1. The sketch of photosentisizer generating ROS species through Type-I or Type-II processes**.**

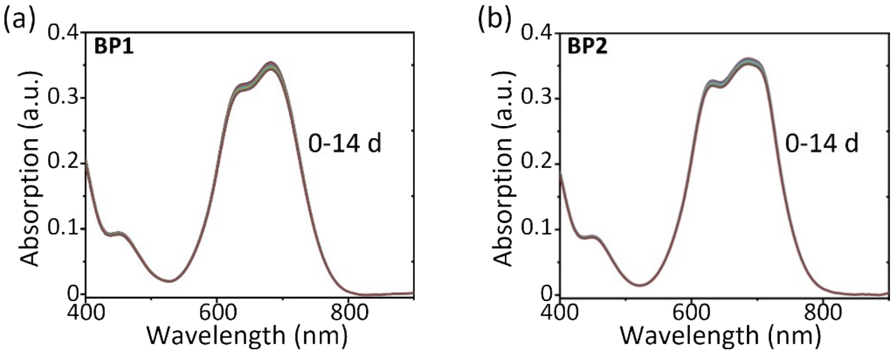

Figure S2. Absorption variation of (a) **BP1-NP** and (b) **BP2-NP** in PBS buffer at room temperaute during 14 days (interval 2 d, c = 10 μM)

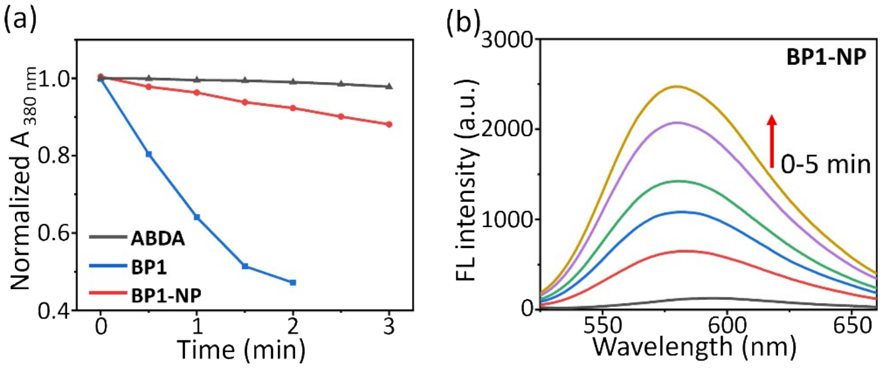

Figure S3. (a) Absorption variations of ABDA in DMF (grey), DMF solution of BP1 (blue) and PBS dispersion of **BP1-NP** (red) under 660 nm light irradiation (P = 50 mW cm^{-2}) during 2-3 min. (b) Fluorescence spectra of DHE in the PBS buffer of **BP1-NP** under 660 nm light irradiation (P = 50 mW cm^{-2}) for 5 min (c = 10 μM, λ_{ex}(DHE)= 525 nm).

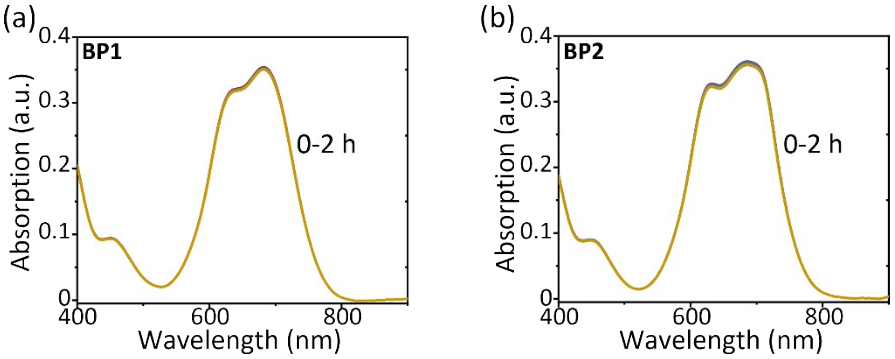

Figure S4. Absorption variation of (a) **BP1-NP** and (b) **BP2-NP** in PBS buffer exposed under 660 nm LED light irradiation (P = 50 mW cm^{-2}) during 2 h (interval 20 min, c = 10 μM)

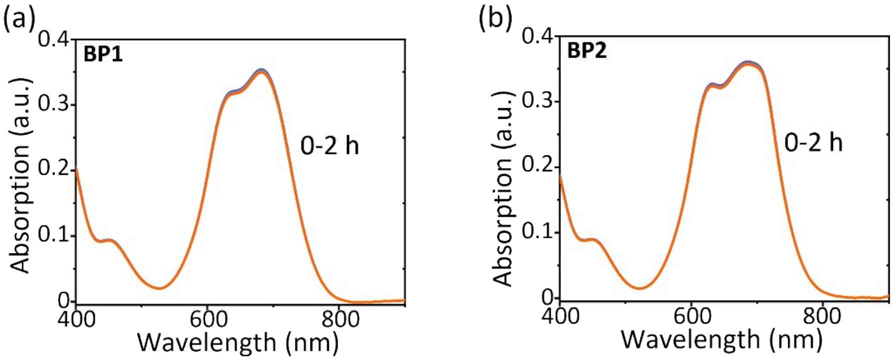

Figure S5. Absorption variation of (a) **BP1-NP** and (b) **BP2-NP** in PBS buffer with H_2O_2 (100 μM) during 2 h (interval 30 min, c = 10 μM)

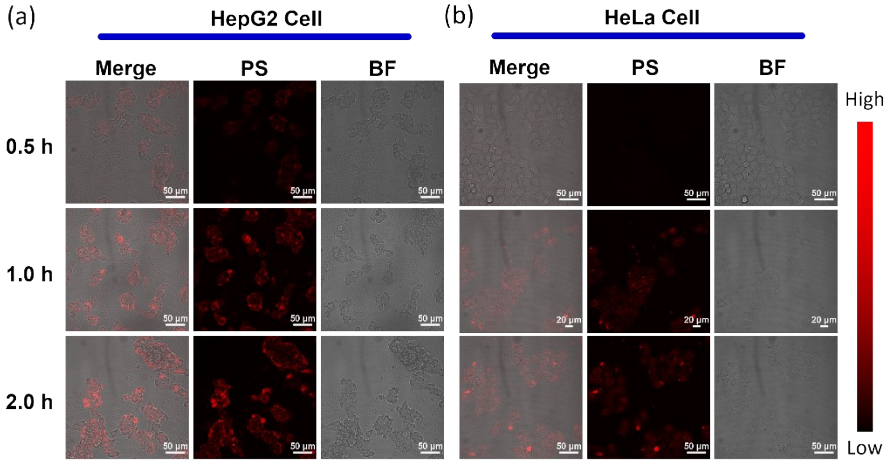

Figure S6. Laser confocal microscope imaging of (a) HepG2 cells and (b) HeLa cells after incubating with **BP2-NP** during different time (c = 4 μM).

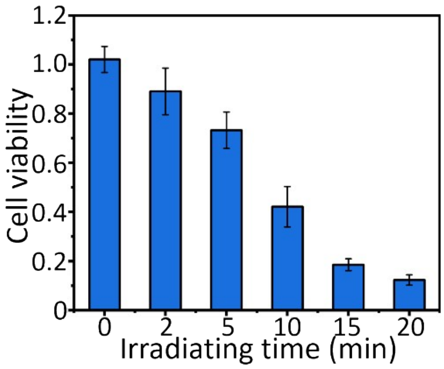

Figure S7. Cell viability of HepG2 cells exposed under 660 light irradiation (*P* = 50 mW cm^{-2}) with different time (0-20 min) after incubating with **BP1-NP** (4 µM).

^{1}H NMR and [13] NMR Spectra

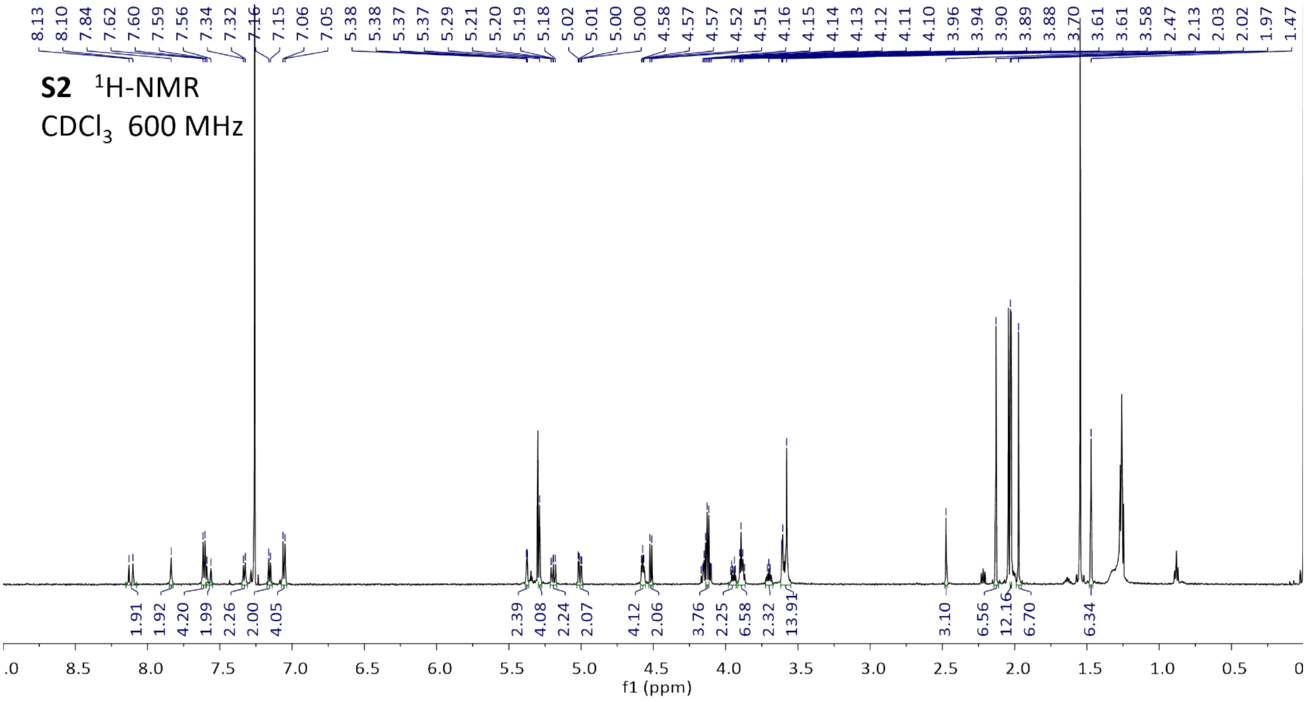

Figure S8. ^{1}H-NMR spectral of compound **S2** in $CDCl_3$.

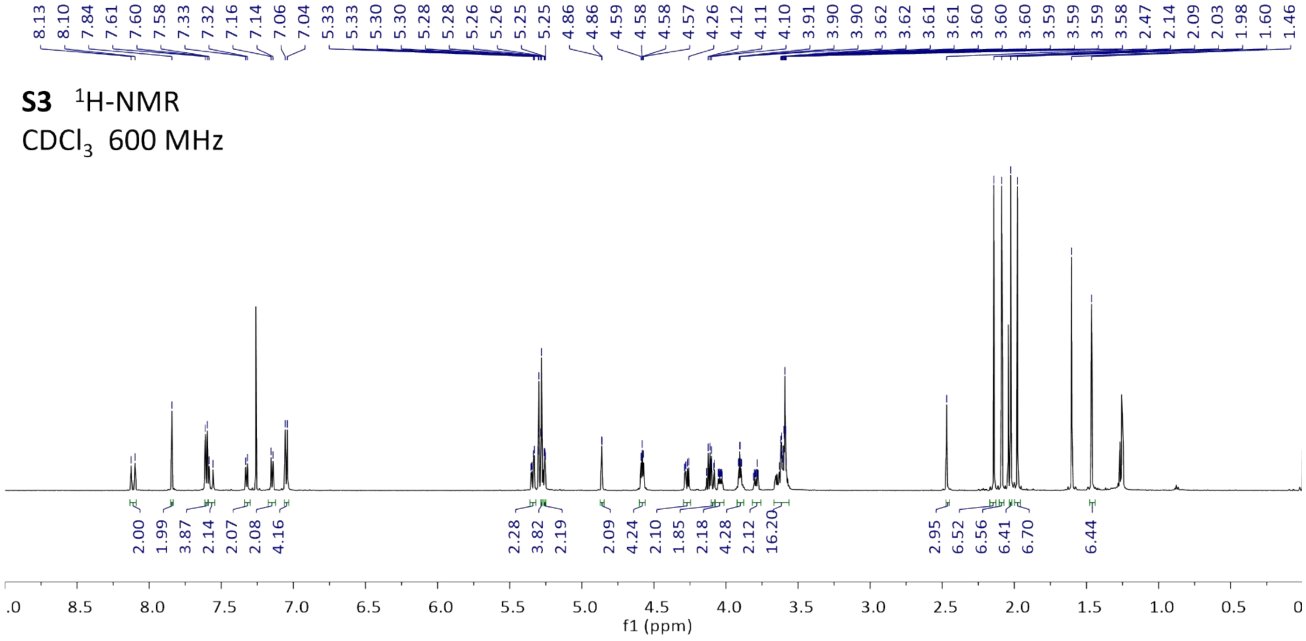

Figure S9. ^{1}H-NMR spectral of compound **S3** in $CDCl_3$.

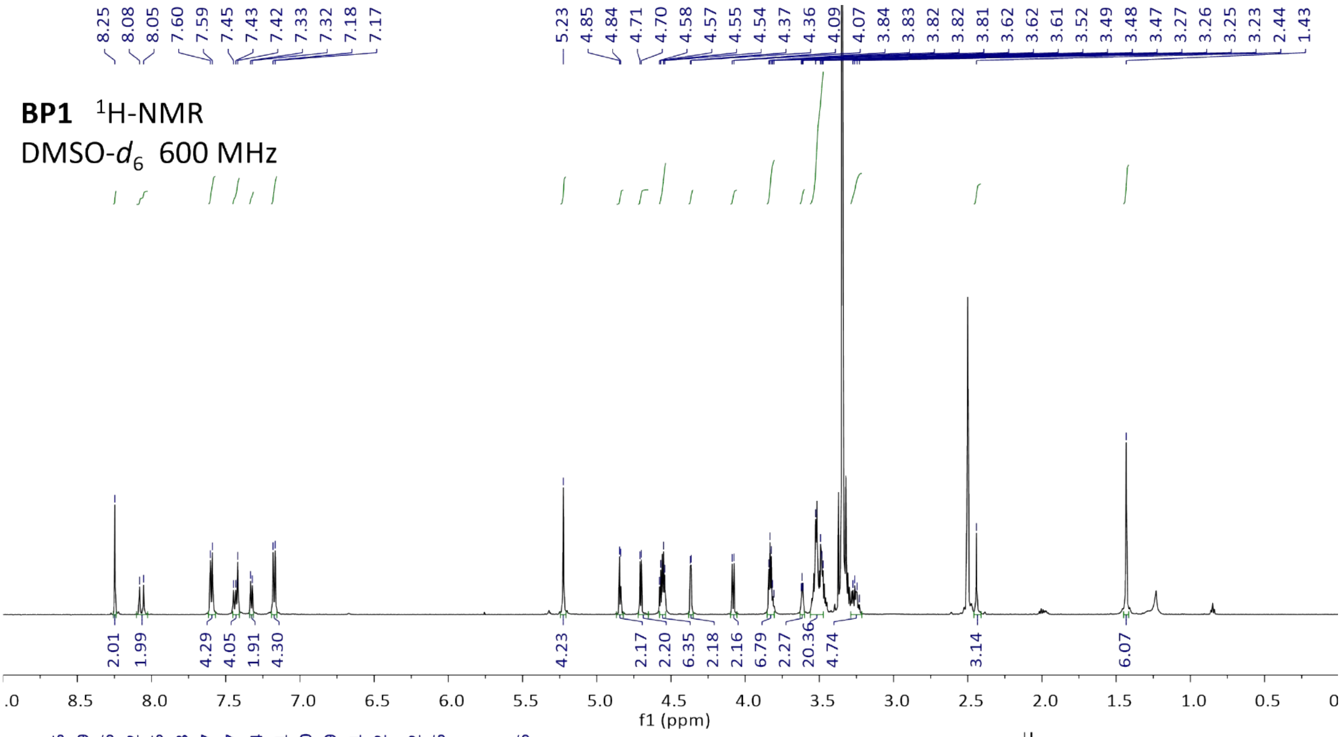

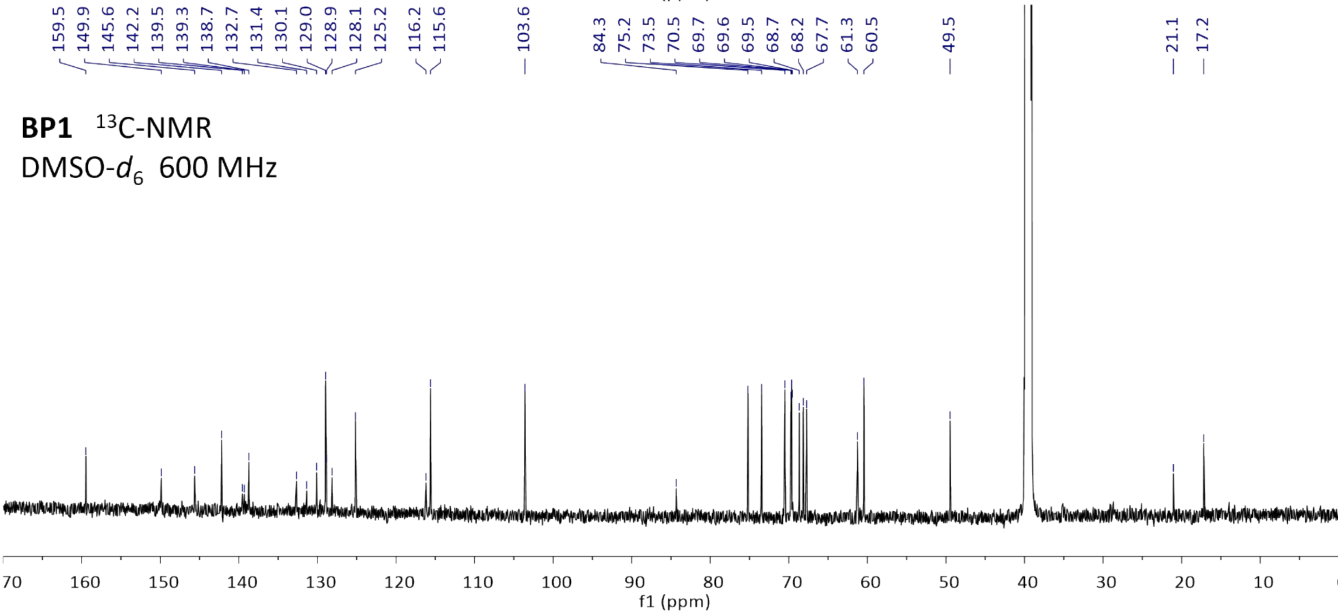

Figure S10. ^{1}H-NMR and ^{13}C-NMR spectra of compound **BP1** in DMSO-d_6.

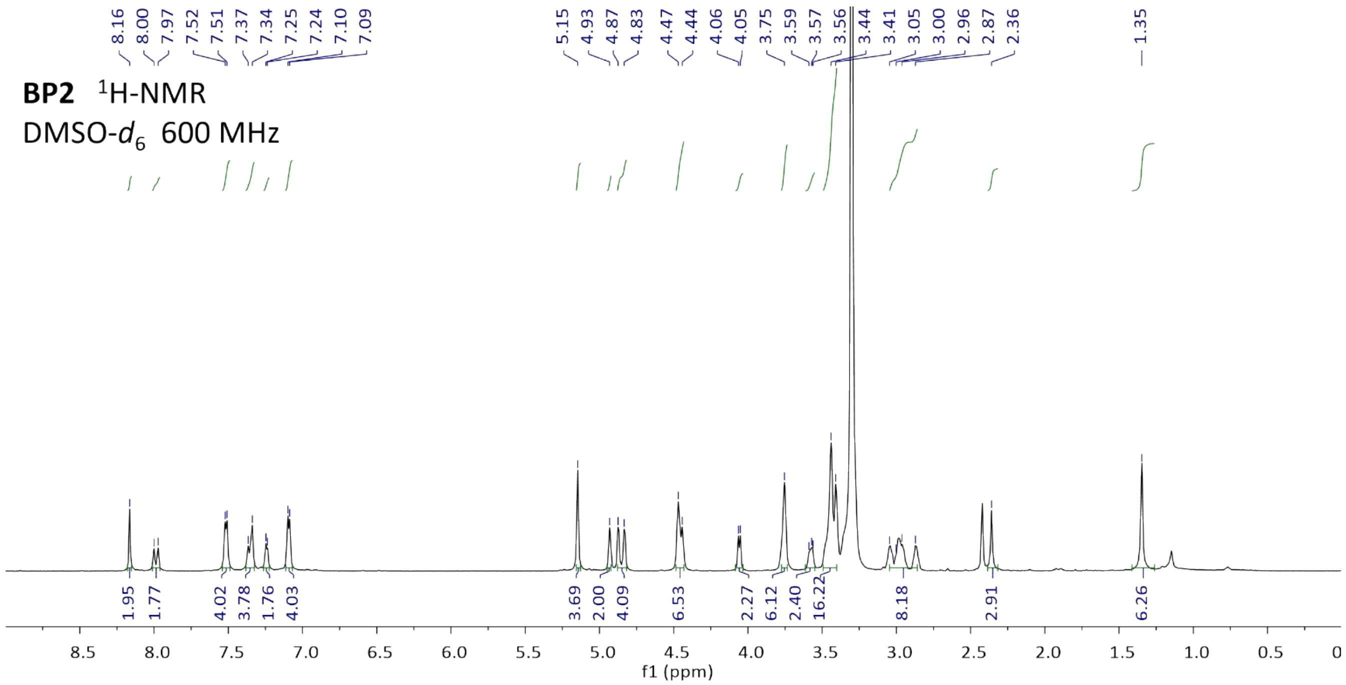

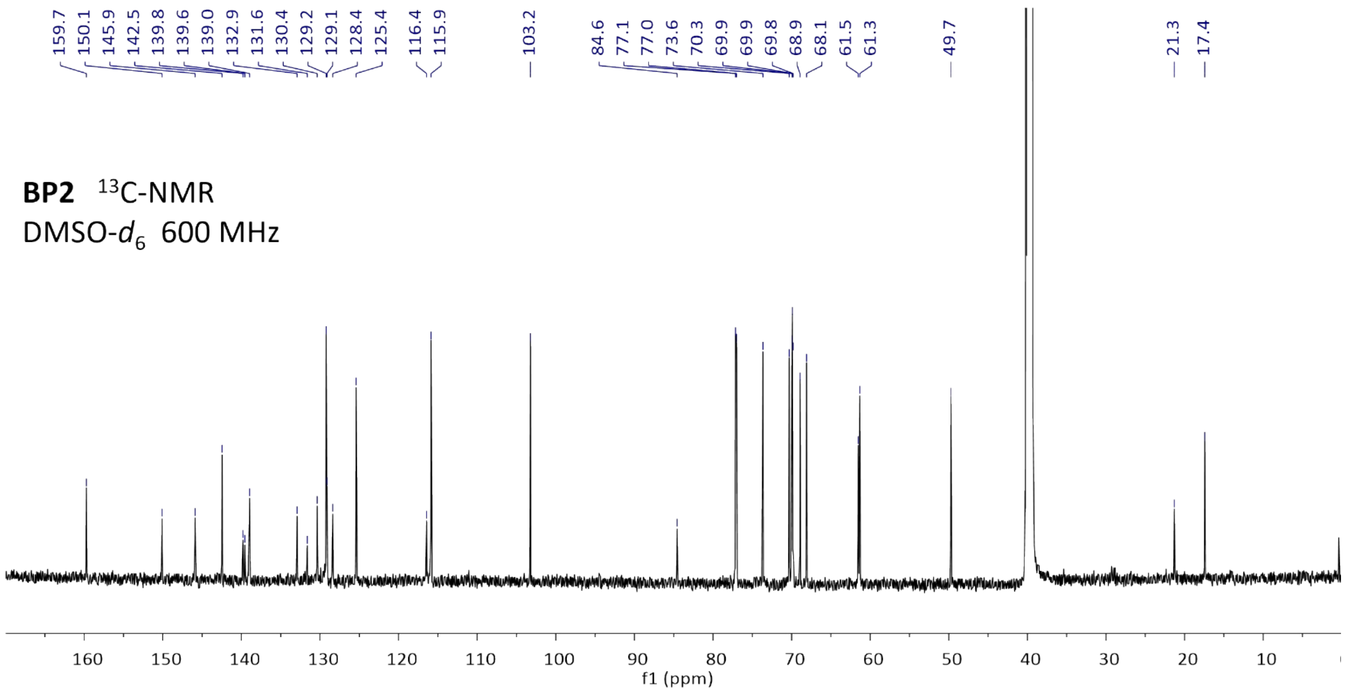

Figure S11. ^{1}H-NMR and ^{13}C-NMR spectra of compound **BP2** in DMSO-d_6.

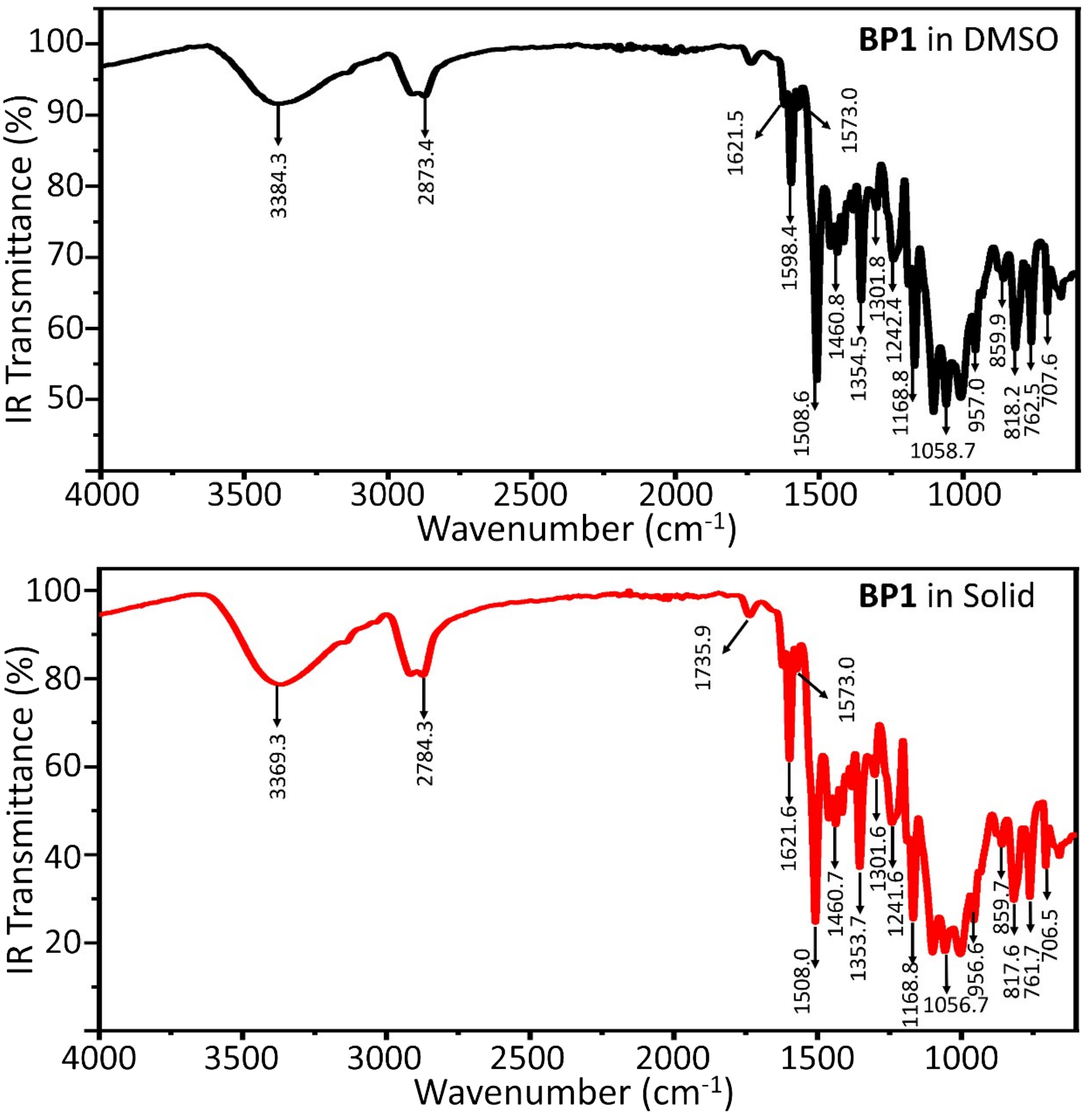

Figure S12. FTIR spectra of compound **BP1** at monomer (black line, in DMSO) and solid (red line) state.

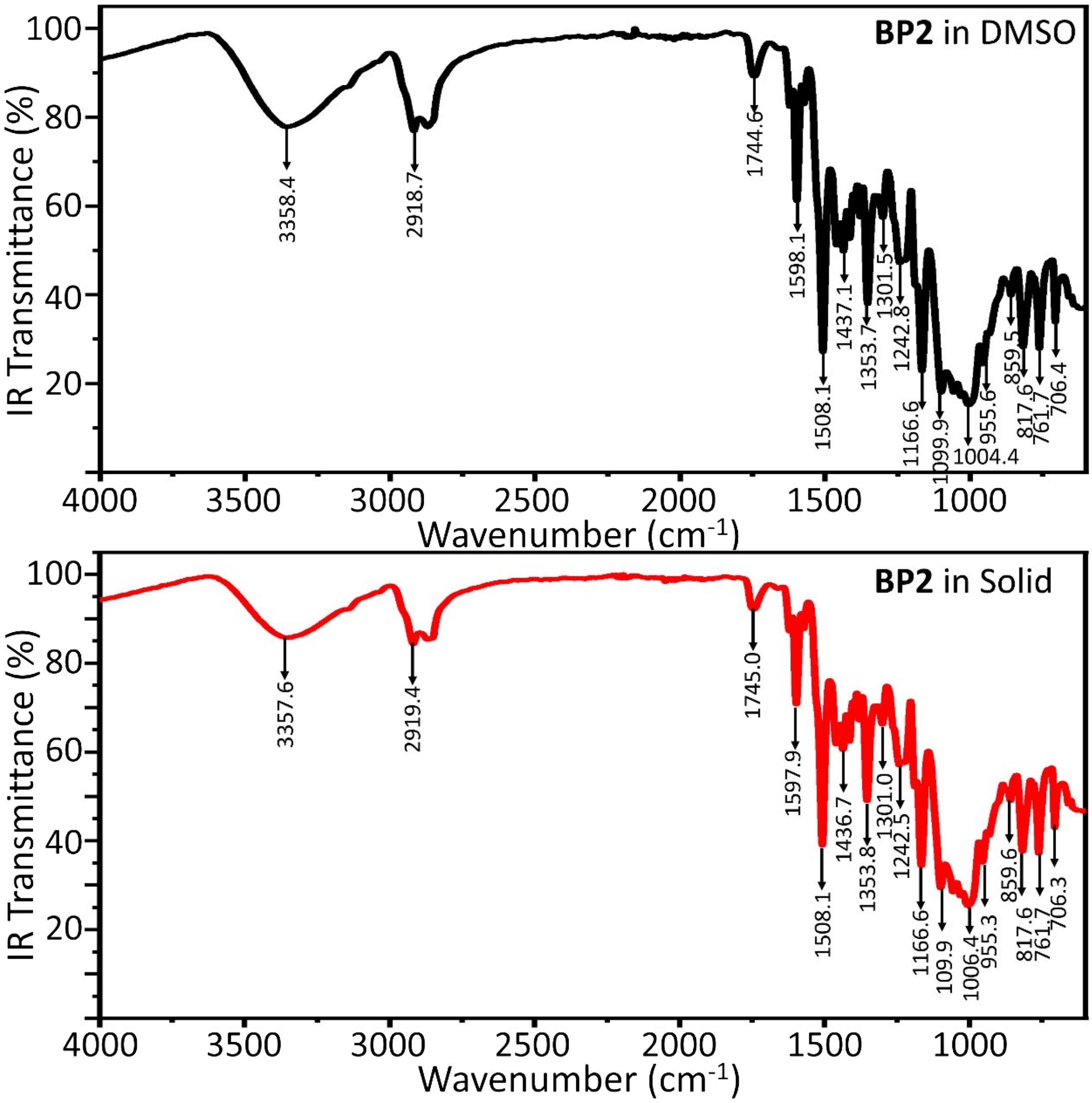

Figure S13. FTIR spectra of compound **BP2** at monomer (black line, in DMSO) and solid (red line) state.

Reference

1. K.-X. Teng, L.-Y. Niu, N. Xie and Q.-Z. Yang, *Nat. Commun.*, 2022, **13**, 6179.
2. D. Serrano-Molina, C. Montoro-García, M. J. Mayoral, A. de Juan and D. González-Rodríguez, J. Am. Chem. Soc., 2022, **144**, 5450-5460.
3. T. Uppal, N. V. S. D. K. Bhupathiraju and M. G. H. Vicente, *Tetrahedron*, 2013, **69**, 4687-4693.
4. L. Dong, M.-Y. Zhang, H.-H. Han, Y. Zang, G.-R. Chen, J. Li, X.-P. He and S. Vidal, *Chem. Sci.*, 2022, **13**, 247-256.
